# Non-Invasive Electrochemical Biosensors for TNF-α Cytokines Detection in Body Fluids

**DOI:** 10.3389/fbioe.2021.701045

**Published:** 2021-09-21

**Authors:** Yang Lu, Qingqing Zhou, Lin Xu

**Affiliations:** ^1^ Department of Cardiovascular, The Second Hospital of Jilin University, Jilin University, Changchun, China; ^2^ State Key Laboratory on Integrated Optoelectronics, College of Electronic Science and Engineering, Jilin University, Changchun, China

**Keywords:** non-invasive, electrochemical biosensor, TNF-α cytokines, cardiovascular disease, diease biomarker

## Abstract

The measurement of pro-inflammatory cytokine tumour necrosis factor-alpha (TNF-α), which is an important indicator of the inflammatory process, has received increasing attention recently because it is easy to extract from body fluid and serves as an early sign of a serious systemic inflammatory disease. Developing fast and simple detection methods to quantify the concentration of TNF-α is essential. Saliva, tears, and urine, which can easily be sampled in a non-invasive way, are considered to be important matrices for monitoring and assessing the physiological status of humans; importantly, they also provide an ideal window for monitoring the concentration of TNF-α. As a fast, accurate, inexpensive, portable, and scalable method, electrochemical biosensors are very promising for biomarker detection in matrices obtained in a non-invasive manner. This review summarises and compares the electrochemical biosensors for the detection of TNF-α in a non-invasive manner and highlights recent advances and future prospects in developing high-performance electrochemical platforms for noninvasive measurement of TNF-α.

## Introduction

Tumour necrosis factor alpha (TNF-α), a pleiotropic cytokine produced by activating monocytes/macrophages in the inflammatory process, is considered an important biomarker of autoimmune diseases (Arya et al., 2007; Ali et al., 2011; Altintas et al., 2014; Kalliolias and Ivashkiv, 2016). It has distinct roles in modulating a series of biological processes, such as in rheumatoid arthritis, human immunodeficiency virus infection, Alzheimer’s disease, stroke, diabetic retinopathy (Boss et al., 2017), and colon and lung cancers (Sadighbayan et al., 2019). It is also related to the severity of cardiovascular lesions and is involved in suppressing myocardial contractility, elevating the content of cardiac peroxynitrite and nitric oxide, changes in intracellular calcium homeostasis, and induction of pathological changes in the failing myocardium (Berthonneche et al., 2004). Moreover, the TNF-α cytokine plays a role in preventing bone and cartilage damage and assisting wound healing (Lipsky et al., 2000; Sandborn et al., 2007). Therefore, developing an accurate and fast TNF-α measurement technique is highly desirable for predicting, preventing, assessing, and monitoring inflammation.

Over the past few decades, evaluating physiological status, detecting illness at an early stage, monitoring disease progression, and assessing therapeutic outcomes through non-invasive methods has become the most ideal and promising approach for clinical diagnosis and research (Bellagambi et al., 2020; Loo and Pui, 2020; Vozgirdaite et al., 2021). Previous studies have revealed that TNF-α can be easily extracted from non-invasively obtained body fluids, such as saliva, tears, and urine (Brennan and Galvin, 2018; Yanez-Sedeno et al., 2019). Monitoring the trace concentration of TNF-α in these body fluids provides a painless, simple, real-time means for cardiovascular disease diagnosis; in particular, it may serve as a sign of inflammation, which is important for the early diagnosis of some acute diseases, such as acute heart failure. However, the level of TNF-α in non-invasively obtained body fluids is extremely low. For example, the TNF-α concentration in the saliva of healthy humans is lower than 3 pg ml^−1^ and increases to about 30 pg ml^−1^ in the saliva of patients with severe autoimmune diseases (Liu and Duan, 2012; Bellagambi et al., 2017). The concentration of urinary TNF-α was determined to be 4 pg mg^−1^ in healthy individuals, normalised by the concomitant urinary creatinine level (to compensate for the TNF-α variations in urine fluid) (Bellagambi et al., 2017). Its concentration can be up to 196.9 ± 121.2 pg/ml in tear fluid, and research is beginning to focus on how immune and inflammatory diseases affect the content of TNF-α in tear fluid, which has a less complex biological composition (Comoglu et al., 2013; Costagliola et al., 2013). Therefore, methods offering low limits of detection and high sensitivity are of great interest for clinical diagnosis and research.

For this purpose, intensive research on TNF-α determination has been conducted, using techniques such as enzyme-linked immunosorbent assay (ELISA), radio immunoassays, chemiluminescence imaging, mass spectrometry, fluorescence immunoassay, and electrochemical immunosensors (Bosnjakovic et al., 2012). Among these techniques, electrochemical biosensors are considered promising platforms that take into account the advantages of easy miniaturisation, small sample volume, simple operation, and low cost (Arya and Bhansali, 2011; Baraket et al., 2016; Povedano et al., 2018). In addition, compared to the ELISA method, which is commonly used in clinical analysis, electrochemical biosensors are more flexible and time-saving, contributing to the rapid evaluation of inflammatory TNF-α (Baydemir et al., 2016). This mini-review summarises the recent progress in the development of electrochemical biosensors for TNF-α determination in a non-invasive manner, and highlights recent advances and future prospects in developing high-performance electrochemical platforms for non-invasive determination of TNF-α biomarkers.

## Non-Invasive Electrochemical Biosensors for Salivary TNF-α

### Immunosensors for Detection of TNF-α in Saliva

As a promising tool for non-invasive detection, the saliva matrix can comprise more than 25% of the proteins in the human serum with strong correlations. Benefiting from the characteristics of easy collection and storage as well as good stability, saliva detection can effectively decrease the demand for cost, sophisticated instruments, and professional operators. In this section, recent studies focused on the detection of salivary TNF-α are summarised, and their advantages and disadvantages are discussed.

**FIGURE 1:**
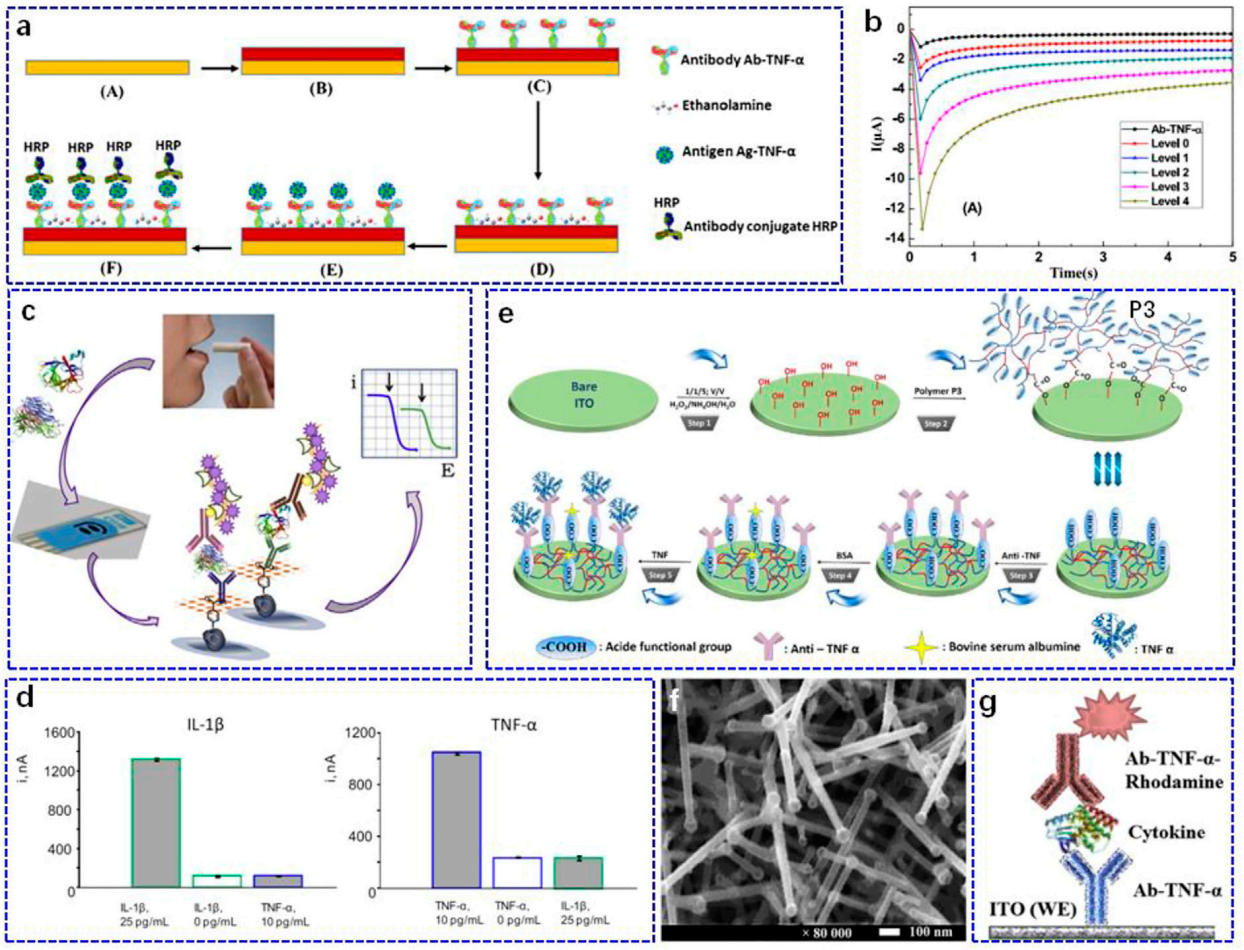
**(A)** Schematic illustration of the immunosensor preparation: A. bare gold, B. electro-addressing of 4-carboxymethylaniline (CMA) on gold working electrode followed by CMA activation, C. biofunctionalisation through incubation with Ab-TNF-α, D. deactivation of the remaining active carboxylic acid groups by incubation in ethanolamine solution, E. incubation of the device in Ag-TNF-α solution, F. subsequent incubation in Ab-TNF-α-HRP solution. **(B)** Chronoamperometric analyses for different incubation of the immunosensor in real human saliva. Reproduced with permission from Barhoumi et al.(2018). Copyright 2018, Elsevier Science SA. **(C)** Schematic illustration of the electrochemical immunosensor for simultaneous determination of TNF-α and IL-1β using dual SPEs. **(D)** Amperometric responses recorded with the dual immunosensor for right: 25 or 0 pg ml^−1^ IL-1β (green), or 10 pg ml^−1^ TNF-a (blue) (right), and left: for 10 or 0 pg ml^−1^ TNF-a (blue), or 25 pg ml^−1^ IL-1β (green). Reproduced with permission from Sanchez-Tirado et al., 2017). Copyright 2017, Elsevier. **(E)** Schematic illustration of the immunosensor based on ITO thin films covered by P3-conjugated polymer. Reproduced with permission from Aydin et al., 2017). Copyright 2017, Elsevier Advanced Technology. **(F)** Surface characterisation of the nanostructured electrode by scanning electron microscopy. **(G)** Schematic illustration of sandwich-type detection for nanostructured ITO electrode-based electrochemical immunosensors. Reproduced with permission from Pruna et al., 2018a). Copyright 2018, Pergamon-Elsevier Science Ltd.

The immunosensor technique with different sensing receptors and transducers is considered as a critical method in biomarker screening (Raluca-Ioana et al., 2000; Baraket et al., 2012) and is considered to be the most promising candidate to compete with well-established laboratory techniques. Barhoumi et al. (2018) designed an electrochemical immunosensor on a gold working electrode (Figure 1A). After modifying 4-carboxymethylaniline (CMA) via electrodeposition, the surface of the electrode was activated by ethanolamine, and then the anti-TNF-α antibody was further functionalised through the carboxylic acid groups of CMA. For TNF-α determination, a secondary antibody (Ab-TNF-α-HRP) labelled with horseradish peroxidase (HRP) was employed to utilise a sandwich-type strategy in tetramethylbenzidine solution. Since the concentration of TNF-α showed a good correlation with heart failure and its severity, the sensor was applied to detect different levels of Ag-TNF-α in artificial saliva. The as-fabricated biosensor exhibited a linear detection range of 1–30 pg ml^−1^ with a fast response in 5 s to the Ag-TNF-α cytokine (Figure 1B). In addition, it showed good selectivity against other cytokines, such as interleukin (IL)-10 and the hormone cortisol. The above sensing performance of the as-built biosensor enables it to serve as a potential tool for fast and accurate quantification of Ag-TNF-α biomarkers. However, the sensing performance of this biosensor was evaluated in artificial saliva, and further clinical examinations should be performed. Similarly, a bifunctional electrochemical immunosensor for the detection of TNF-α and IL-1β cytokines was reported by Sanchez-Tirado et al. (2017). In this study, they used screen-printed carbon electrodes (SPCEs) with dual working electrodes as the sensing platform and then introduced CMA as scaffolds for further modification of the corresponding antibodies (Figure 1C). Such a dual immunosensor displayed a linearity range of 1–100 pg ml^−1^ with a limit of detection (LOD) of 0.85 pg ml^−1^ and 0.5–100 pg ml^−1^ with an LOD of 0.38 pg ml^−1^ for TNF-α and IL-1β, respectively, which was sufficient for application in clinical testing. In addition, the cross-talk between the adjacent SPCEs that were modified with anti-IL or anti-TNF in the dual immunosensor was non-perceptible, and no obvious differences were detected in the amperometric responses of immunosensors when faced with the non-target antigen or without the corresponding target antigen (Figure 1D). When tested in the saliva sample, the results obtained from the dual electrochemical immunosensor showed almost consistent results with the clinical data measured from the ELISA arrays. Note that the reagent consumption was also very low (2.5 μl) compared to that of ELISA (100 μl).

Both of these immunosensors used CMA as the conjugated and blocking layer; however, the weak electron transfer capacity limits the sensing performance to some extent (Barhoumi et al., 2019). To avoid this problem, Aydin et al. (2017) developed a label-free immunosensor for salivary TNF-α determination by applying a semiconductive polymer (poly (3-thiophene acetic acid, P3)) on an indium tin oxide (ITO) electrode (Figure 1E). Polymer P3 not only showed good conductivity but was also rich in carboxylic acid groups, which was beneficial for the effective immobilisation of the anti-TNF-α antibodies. Using P3 as the conjugated matrix, the linear detection response of the developed immunosensor had an LOD of 3.7 fg ml^−1^, which was suitable for clinical salivary sample determination. To evaluate the practicability of the P3-based electrochemical immunosensor, it was used to quantify the level of TNF-α in clinical saliva from a local hospital, and it showed recoveries between 98.39 and 105.20%, demonstrating the good potential of the developed immunosensor to be employed as an accurate diagnostic tool for TNF-α determination. Using a similar strategy, the sensing performance of the TNF-α immunosensor could be improved by using a polymer-coated magnetic microparticles matrix for antibody immobilisation (Barhoumi et al., 2019).

Although the above polymer-coated immunosensors could realise the improvement of the sensing properties, the employed electrode-modified strategy still requires polymers or other layers to cover the whole surface (Eletxigerra et al., 2015; Arya and Estrela, 2020; Nessark et al., 2020), which may require additional nanostructures to maintain a larger surface area (Hou et al., 2013; Baraket et al., 2017; Yola and Atar, 2021). For this purpose, Pruna et al. (2018a) constructed a nanostructured ITO electrode with a high surface-to-volume ratio to build opto-electrochemical sensors (Figure 1F). Together with the good optical transparency and electrical conductivity, a nanostructured ITO electrode was fabricated to sandwich the antibody-antigen type immunosensor for TNF-α recognition (Figure 1G). Because of its good linear range of 10–100 pg ml−^1^, the proposed immunosensor was expected to trigger a stable response to trace TNF-α in salivary samples from healthy patients.

### Other Electrochemical Biosensors

**FIGURE 2:**
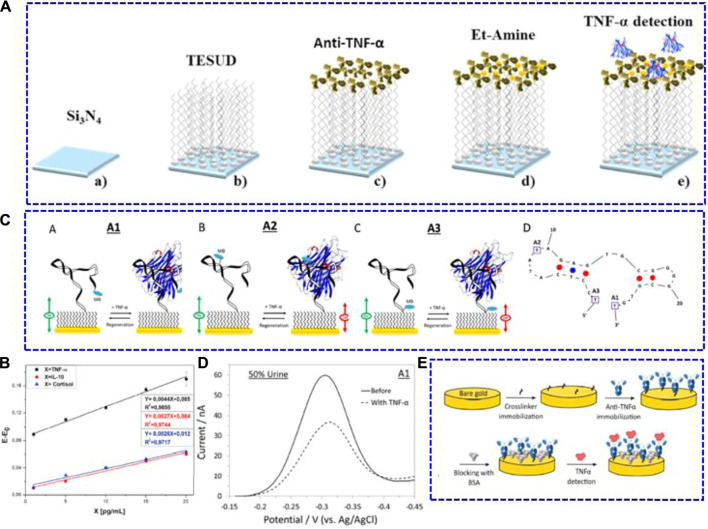
**(A)** Schematic illustrations of the chemical surface modification and biofunctionalisation process of the immunosensor with anti-TNF-α antibodies. a) S_i3_N_4_ surface cleaning and activation, **(B)** S_i3_N_4_ surface functionalisation with TESUD, **(C)** anti-TNF-α antibodies immobilisation step, d) S_i3_N_4_ surface blocking using 1% ethanolamine, and **(E)** electrochemical TNF-α detection step. **(B)** Detection sensitivity curves of Ag-TNF-α, IL-10 cytokine, and cortisol in artificial saliva. Reproduced with permission from Bahri et al. (2020). Copyright 2020, Elsevier.**(C)** Schematic illustrations of the three electrochemical aptamer-based TNF-α biosensors (A–C) and the optimal (minimum energy) structure of the adopted aptamer (D). **(D)** Alternating current voltammetry of the A1 sensor in the absence and presence of 100 nM TNF-α in a 50% synthetic urine sample. Reproduced with permission from Mayer and Lai (2018) Copyright 2018, Elsevier.**(E)** Schematic representation of the immunosensor biofunctionalisation process. Reproduced with permission from Cruz et al. (2019). Copyright 2019, American Chemical Society.

Apart from the typical amperometric method, electrochemical biosensors based on the principle of capacitance as well as biosensors with aptamers as recognition species have also been developed for TNF-α in saliva. Bahri et al. (2020) studied a capacitance electrochemical biosensor using a silicon nitride (Si_3_N_4_) transducer (Figure 2A). After activation by the piranha solution, silanol and silylamine groups were introduced on the surface of the Si_3_N_4_ transducer in conjunction with bio/chemical substances. Then, a self-assembled monolayer of aldehyde-silane (11-(triethoxysilyl) undecanal) (TEUSD) was further modified for anti-TNF-α bonding, and the bonded condition was checked using the micro-contact printing technique. The sandwich antibody-antigen bio-recognition was chosen for TNF-α determination, in which the anti-TNF-α antibody was labelled with rhodamine. Mott-Schottky analyses of this capacitance electrochemical biosensor were used for TNF-α quantification. The result showed that this biosensor had a sensitivity of 4.4 mV pM^−1^ with an LOD of 1 pg ml^−1^ in artificial saliva (Figure 2B). Furthermore, it displayed good selectivity against cortisol and IL-10.

Aptamers are single-stranded DNA or RNA molecules that can bind to specific targets in terms of base pairing (Tuerk and Gold, 1990; Ellington and Szostak, 1992). Unlike antibodies utilised in immunosensors, biosensors using aptamers as biorecognition components are more stable under different experimental conditions and are much cheaper than the generation of antibodies. Mayer and Lai. (2018) studied electrochemical aptamer-based TNF-α biosensors using three aptamer probes labelled with methylene blue (Figure 2C). When exposed to TNF-α, the conformation of the chosen aptamer probe changed, which adjusted the electron transfer capacity of the labelled methylene blue. As a result, the corresponding redox current was altered. Among these biosensors, the one employing the aptamer with methylene blue modified at the distal end (A1 biosensor, as shown in Figure 2C) had the best sensing performance. This biosensor showed an LOD of 100 pM and good reproducibility. Moreover, it exhibited a good response in synthetic fluid with 50% urine and 50% saliva (Figure 2D).

In addition to saliva, electrochemical biosensors for TNF-α in other non-invasively obtained body fluids, such as tears, were also studied. Cruz et al. (2019) built a highly sensitive label-free immunosensor for TNF-α screening (Figure 2E). The SPE (screen-printed electrodes) with gold as the working part was modified with sulfo-LC-SPDP (sulfosuccinimidyl 6-(3’-(2-pyridyldithio) propionamido) hexanoate) as a crosslinker for anti-TNF-α immobilisation. After blocking with bovine serum albumin, the biosensor was further bonded with TNF-α for quantitative determination. The sensing performance of the as-built immunosensor was tested using Nyquist plots. As calculated, it exhibited an LOD of 0.085 pg ml^−1^ in PBS. To investigate the practical applicability in clinical samples, the immunosensor was applied to measure the trace concentration of TNF-α in tears, blood serum, and human cerebral spinal fluid. As a result, the immunosensor could detect TNF-α in tears beyond the LOD of ELISA (4 pg ml^−1^).

### Smart Point-of-Care Biosensors

**FIGURE 3:**
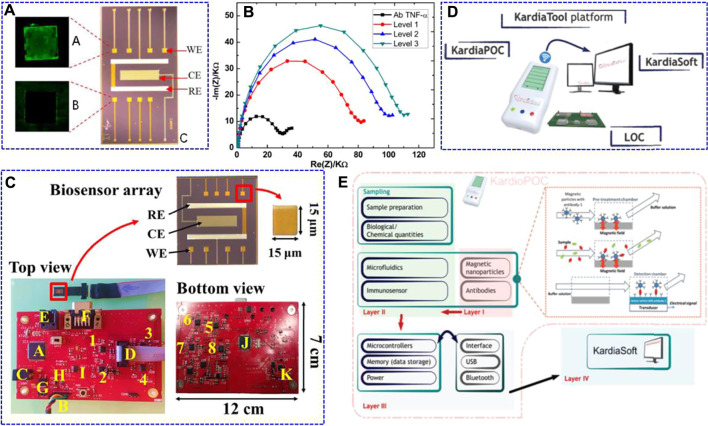
Fluorescence optical images of A: anti-TNF-α/TNF-α-modified working electrode (WE) after incubation in secondary fluorescent TNF-α, B: no functionalised WE, and C: optical image of the fully integrated BioMEMS. WE: working electrode, CE: counter electrode, RE: reference electrode. Reproduced with permission from Longo et al. (2016). Copyright 2016, Elsevier Science BV. **(B)** Nyquist impedance plots obtained from the standard addition method performed on a real saliva sample (black point) Ab TNF-α for Ag TNF-α (red point) Level 1 (corresponding to an addition of 0 pg ml^−1^); (blue point) Level 2 (addition of 3 pg ml^−1^); (green point) Level 3 (addition of 7 pg ml^−1^). Reproduced with permission from Bellagambi et al. (2017). Copyright 2017, Elsevier Science SA. **(C)** Manufacturing details of the printed circuit board and the biosensor array. The potentiostatic circuits for the different channels are labelled as 1–4 (top view) and 5–8 (bottom view). Regarding the rest of the elements, A) is the PIC32 microcontroller; B) is the UART connector for PC communication; C) is the external power supply; D) is the 10-pin connector for the biosensor array; E) is the RJ-11 connector to program the microcontrollers; F) is the data bus; G) is the LM1117 voltage regulator; H) is the MCP1603 B voltage regulator; I) is the ADR4525 stable voltage reference; J) is the PIC24 microcontroller, and K) is the TC962 voltage regulator. Reproduced with permission from Pruna et al. (2018b). Copyright 2018, Elsevier Advanced Technology. **(D)** KardiaTool concept. **(E)** High level architecture of the KardiaTool platform. Reproduced with permission Tripoliti et al. (2018). Copyright 2018, IEEE Engineering in Medicine and Biology Society.

With the rapid development of technology, the need for intelligent POC devices in clinical and home self-monitoring is rapidly increasing, especially for patients with cardiovascular disease. Longo et al. (2016) constructed an integrated electrochemical bioMEMS for TNF-α cytokine detection. Because the BioMEMS had eight gold working microelectrodes (integrated reference and counter electrodes on one chip), it allowed for the simultaneous detection of multiple target molecules through the antibody-antigen bio-recognition mode (Figure 3A). Using EIS characterisation, the BioMEMS platform exhibited a linear detection range of 1–15 pg ml^−1^ in artificial human saliva against other cytokines, such as IL-1 and IL-8. They further applied the same system for TNF-α determination in human saliva with a linear range of 1–100 pg ml^−1^ and an LOD of 3.1 pg ml^−1^ (Figure 3B) (Bellagambi et al., 2017). Miniaturising the potentiostat in POC electrochemical biosensing systems while maintaining their effective characteristics is still a challenge. Using a similar BioMEMS electrode, Pruna et al. (2018b) integrated analogue circuitry, microcontrollers, and digital filter implementation to avoid the complex structures of the previously reported circuits, which have a total size of 12 × 7 × 2 cm^3^. This electronic system was further connected to BioMEMS electrodes for TNF-α determination (Figure 3C). This device had a high sensitivity in the randomly chosen range of 266 pg ml^−1^–666 ng ml^−1^.

Tripoliti et al. (2018) presented an integrated device with the name of Kardiatool platform as an example for the non-invasive diagnosis of heart failure (Figure 3D). This device consists of two parts: a portable POC device (KardioPOC) and the corresponding software. The KardioPOC has a first layer with magnetic nanoparticles and antibodies for target species recognition, and then an added microfluidic system, immunosensor component, and saliva samples for measurement. The third layer is composed of integrated circuit sections, such as the power supply, memory, microcontroller, and communication module (Figure 3E). In addition, it can simultaneously measure four different biomarkers, such as TNF-α, IL-10, N-terminal pro b-type natriuretic peptide (NT-pro BNP), and cortisol. This platform provides a proof of concept for the POC device for heart failure evaluation. The above work is promising for the development of low-cost, portable, and time-saving electrochemical systems for POC TNF-α determination. However, POC platforms for *in situ* tracking of cardiovascular diseases are not satisfactory, and the development of new types of biosensors is still needed.

## Conclusion

This review summarises the progress in electrochemical biosensors for non-invasive detection of TNF-α cytokines, including immunosensors for detection of TNF-α in saliva, electrochemical biosensors based on other principles or other non-invasive fluids (such as tears), and smart POC biosensors. Among these sensors, saliva testing plays an important role in non-invasive TNF-α cytokine determination because of its ability to be easily collected, good stability, and suitable TNF-α concentration within the detection capability of electrochemical biosensors. These electrochemical biosensors for non-invasive detection of TNF-α cytokines have promising prospects for continuous, fast, real-time, and portable monitoring of cardiovascular disease. Moreover, smart POC biomarkers provide a great solution for chronic disease self-management, such as chronic heart failure. To make a clearer comparison, the sensing performance, such as sensitivity and specificity, of different non-invasive biosensors that discussed in this review are listed in Table 1. As compared, the most immunosensors can response to TNF-α concentration level of pg mL^−1^. Due to the different electrochemical methods, It’s hard to quantitatively compare the sensitivity. However, the selectivity is generally good, which is related to the well-designed structure and specific recognition of Ab-TNF-α. Especially, the Au WE/sulfo-LC-SPDP/Ab-TNF-α immunosensor built by Cruz et al. exhibited a high specificity to TNF-α, which is at least 6 times higher than interferon gamma (IFNγ) and IL-4. This can be attributed to the introduction of SPDP monolayer which is considered to be effective for Ab-TNF-α immobilization.

**TABLE 1:** A comparison of the sensing performance of non-invasive immunosensors discussed in the review.

Immunosensor structure	Sensor type	Selectivity^a^	Sensitivity	Linear range (pg ml^−1^)	LOD (pg ml^−1^)	References
Au WE/CMA/Ab-TNF-α/antigen-Ag-TNF-α/Ab-conjugate-HRP	Chronoampemetric	>3.5	I (μA) = 0.0758C (ppt)-0.2125, *R* ^2^ = 0.993	1–30	1	Barhoumi et al. (2018)
SPCE/DWCNTs/Phe-Ab-TNF/TNF-α/Biotin-Ab-TNF/poly-HRP	Amperometric	>5	I (nA)= (766.3 ± 0.2) logC (pg mL^−1^) + (237.8 ± 0.2) *R* ^2^ = 0.999	1–200	0.85	Sanchez-Tirado et al. (2017)
Si_3_N_4_ ISFET/TESUD/Ab-TNF-α	Electochemical	>2.5	Not mentioned	5–20	5	Vozgirdaite et al. (2021)
ELISA plate/PEG/G4-OH/Ab-MAB610	ELISA	∼3	A (O. D.) = 0.1502 + 0.0037C (pg mL^−1^) R = 0.996	0–300	0.48	Bosnjakovic et al. (2012)
Au WE/CMA/Ab-TNF-α	Potentiostat	>1.3	Not mentioned	266–666,000	266	Pruna et al. (2018b)
ITO/Polymer-P3/NHS-EDC/Ab-TNF-α/BSA	Impedimetric	>2.5	A (kΩ) = 1,381.8C (pg mL^−1^)+54.811, *R* ^2^ = 0.998	0.01–2	0.0037	Aydin et al., 2017
Si_3_N_4_ WE/TESUD/Ab-TNF-α/Et-Amine	Capacitance	>2	Y (mV) = 0.004C (pM) +0.058, *R* ^2^ = 0.9908	1–30	1	Bahri et al. (2020)
Au WE/sulfo-LC-SPDP/Ab-TNF-α	Impedimetric	>6	Y(Ω) = 0.6199C (log (TNF-α, pg mL^−1^)) + 0.02926, *R* ^2^ = 0.9852	1–25	0.085	Cruz et al. (2019)
Au WE/CMA/Ab-TNF-α	Impedimetric	∼1	Not mentioned	1–15	—	Bellagambi et al. (2017)
Au WE/CMA/Ab-TNF-α	Impedimetric	>5	Not mentioned	1–15	—	Longo et al. (2016)
ITO/CMA/Ab-TNF-α	Impedimetric	—	Not mentioned	10–100	5	Pruna et al. (2018a)

a The selectivity is defined as the ratio of response to TNF-α to that of other interferences. WE: working electrode, LOD: limit of detection; CMA:4-carboxymethylaniline; HRP: horseradish peroxidase; SPCE: screen-printed carbon electrodes; DWCNTs: double-walled carbon nanotubes; ISFET: ion-sensitive field effect transistor TESUD: 11- (triethoxysilyl) undecanal ELISA: enzyme-linked immunosorbent assay; PEG: polyethylene-glycol; G4-OH: poly (amidoamine) dendrimer; NHS: N-hydroxysuccinimide; BSA: bovine serum albumin.

Although research attention has been rapidly gained in the area of non-invasive TNF-α cytokine detection via electrochemical biosensors, further optimisation of sensor reliability should be critically investigated, and biosensors that can reliably and accurately determine TNF-α in clinical settings are still in their infancy. Besides the TNF-α cytokine, lactate, 8-isoprostalandin F_2α_, C-reactive protein, and IL-1β from saliva can also act as biomarkers for cardiovascular disease (Aydın et al., 2018; Vilian et al., 2019; Ghimenti et al., 2020). Designing biosensors that can simultaneously detect multiple biomarkers is necessary to improve accuracy and reliability. Owing to the accessibility of non-invasive body fluids, especially saliva, the design of portable and integrated devices for POC applications in remote and self-monitoring is promising, and related studies are needed.
